# Development and evaluation of an inactivated coxsackievirus A16 vaccine in gerbils

**DOI:** 10.1080/22221751.2022.2093132

**Published:** 2022-08-11

**Authors:** Yi-Sheng Sun, Yong Xia, Fang Xu, Hang-Jing Lu, Zi-An Mao, Meng Gao, Tian-Yuan Pan, Ping-Ping Yao, Zhen Wang, Han-Ping Zhu

**Affiliations:** a Key Lab of Vaccine, Prevention and Control of Infectious Disease of Zhejiang Province, Zhejiang Provincial Center for Disease Control and Prevention, Hangzhou, People’s Republic of China; b Zhejiang Pukang Biotechnology Co., LTD., Hangzhou, China; c Department of General Medicine, The First Affiliated Hospital, Zhejiang University, Hangzhou, People’s Republic of China

**Keywords:** Coxsackievirus A16, MRC-5 cell, inactivated vaccine, gerbil, immunization

## Abstract

Coxsackievirus A16 (CVA16) is one of the major pathogens responsible for human hand, foot, and mouth disease (HFMD), which has threatened the health of young children, particularly in Asia-Pacific nations. Vaccination is an effective strategy for protecting children from CVA16 infection. However, there is currently no licensed CVA16 vaccine for use in humans. In this study, we isolated a high-growth CVA16 virus strain in MRC-5 cells and developed an MRC-5-adapted vaccine candidate strain termed CVA16-393 via two rounds of plaque purification. The CVA16-393 strain was grouped into the B1b subgenotype and grew to a titre of over 10^7^ TCID_50_/ml in MRC-5 cells. The VP1 gene region of this strain, which contains the major neutralizing epitopes, displayed high stability during serial passages. The inactivated whole-virus vaccine produced by the CVA16-393 strain induced an effective neutralizing antibody response in *Meriones unguiculatus* (gerbils) after two doses of intraperitoneal inoculation. One week after the booster immunization, the geometric mean titres of the neutralizing antibodies for the 10246, 40812TXT, 11203SD, TJ-224 and CA16-194 strains from different regions of China were 137.8, 97.8, 113.4, 64.1 and 122.3, respectively. A CVA16 vaccine dose above 25 U was also able to provide 100% cross-protection against lethal challenges with these five clinical strains in gerbils. Immunization at a one-week interval could maintain a high level of neutralizing antibody titres for at least 8 weeks. Thus, the vaccine produced by this CVA16-393 strain might be promising.

Coxsackievirus A16 (CVA16) is a non-enveloped virus belonging to the genus *Enterovirus* of the family *Picornaviridae* [1]. CVA16 and enterovirus 71 (EV-A71)*,* are the two major pathogens of HFMD. Before 2008, HFMD was rarely regarded as an epidemic infectious disease in China. A large epidemic wave of HFMD occurred in Fuyang city, and it rapidly developed into a national-scale epidemic, covering 28 provinces within 3 months and with 345,159 reported cases in 2008 [2]. Since then, a national surveillance system for HFMD has been used in China [1,3]. In the last decade, HFMD has become one of the most common infectious diseases in China. With the use of the EV-A71 vaccine since late 2016, the number of EV-A71-related HFMD patients has markedly decreased [1]. However, the CVA16 epidemic continues today, and more than 22% of all HFMD cases are reported each year in China [4–6]. Thus, CVA16 is a major public health problem.

HFMD caused by CVA16 infection is generally self-limited and tends to be mild, presenting with fever, skin rash, mouth ulcers and pharyngitis. Occasionally, CVA16 can cause serious infections with complications, including encephalitis, myocarditis and acute flaccid paralysis [7–9]. Vaccine is one of the most effective measures for controlling virus spread and infection. At present, no human CVA16 vaccine is available for human use. The development of a safe and effective vaccine remains an urgent priority. Cell culture-based vaccine production systems, which allow a rapid and simple scaled-up platform, have been widely used [10]. Several mammalian cell lines, including an African green monkey kidney cell line (Vero) and a human diploid cell line (MRC-5), have been used in vaccine production. MRC-5 cells, which originated from the human embryonic lung tissue and exhibit less risk of heterologous virus infection than Vero cells [11], are suitable for human CVA16 vaccine production. In this study, we isolated a clinical strain, named CVA16-393, from a swab sample by inoculation into MRC-5 cells. We performed as many as 20 serial passages of CVA16-393 along with two rounds of plaque purification in MRC-5 cells. The growth and genetic stability of the virus as a vaccine candidate strain were assessed. Additionally, the efficacy of an inactivated CVA16 vaccine based on the CVA16-393 strain was detected using a gerbil model. The cross-protection induced by the CVA16 vaccine in gerbils was evaluated, and the optimal immunization procedure was determined. Through these experiments, we demonstrated that the CVA16-393 virus strain might be a suitable one for vaccine production in MRC-5 cells.

## Methods

### Ethical approval

Gerbils and BALB/c mice were obtained from the Animal Center of Zhejiang Academy of Medical Sciences (Hangzhou, China), and all animal experiment protocols were approved by the animal ethics committee of Zhejiang Academy of Medical Sciences. The experiments were performed in accordance with the principles of the Declaration of Helsinki.

### Viruses and cells

The CVA16-393 strain (GenBank ID: KY014077) was isolated from swab samples of a 15-month-old boy with HFMD in the Hangzhou Sixth People’s Hospital (Hangzhou, China) in 2008. Swab samples were pretreated with 1000 units/ml of penicillin and 1000 μg/ml of streptomycin (Sigma, St. Louis, MO, USA) for 1 h and then inoculated into MRC-5 cells to yield the primary viral isolate. CVA16-194 (GenBank ID: KX056216, genotype B1b) was described previously [12]. Four strains, namely, 11203SD (from Shandong, China, 2008, genotype B1b), 10246 (from Zhejiang, China, 2009, genotype B1a), TJ-224 (from Tianjin, China, 2010, genotype B1a) and 40812TXT (from Jiangsu, China, 2012, genotype B1b) were provided by Sinovac Biotech Ltd. (Beijing, China). These virus stocks were grown in Vero cells in Minimum Essential Medium (MEM, Gibco, NY, USA) at 35 °C. At 3 days post-infection, when 80% of the cells showed typical cytopathic effects (CPEs), the virus stocks were harvested via three freeze–thaw cycles of infected Vero cells. The virus stocks were stored at −80 °C, and the 50% tissue culture infective dose (TCID_50_) of the virus in Vero cells was calculated using the Reed and Muench method as described previously [13]. MRC-5 and Vero cells were grown in MEM supplemented with 10% heat-inactivated fetal bovine serum (FBS, Every Green, Hangzhou, China), 100 units/ml of penicillin, and 100 μg/ml of streptomycin (Solarbio, Beijing, China). All the cells were cultured in an incubator at 37 °C with 5% CO_2_.

### Phylogenetic analysis

Total RNA from the virus was isolated using a QIAamp Viral RNA Mini Kit (Qiagen, Hilden**,** Germany). VP1 gene segments were amplified by RT-PCR using specific primer sets: CVA16-VP1-F, 5’-ATTGGTGCTCCCACTACAGC-3’ and CVA16-VP1-R, 5’-GCTGTCCTCCCACACAAGAT-3’ [14]. Amplification of the viral regions was performed using a One-step RT–PCR Kit (Takara, Shiga**,** Japan). The VP1 gene (891 nucleotides) sequences of the six CVA16 strains used in this study were compared with reference strains from the Asia-Pacific region. A phylogenetic tree was constructed using the neighbor-joining method with MEGA software (version 5.1).

### Establishment of the CVA16 seed stock in MRC-5 cells and detection of the stability of CVA16-393

The CVA16-393 strain was subjected to two rounds of plaque purification in MRC-5 cells and then manipulated to obtain the master virus seed stock CVA16-393-p5 (P5) and the working virus seed stock CVA16-393-p7 (P7). Briefly, primary viral isolates of CVA16-393 were inoculated at 10-fold dilutions in MRC-5 cells to obtain the plaque clone CVA16-393-p2 (P2). P2 was then used to obtain the second round of purified plaque (CVA16-393-p3) via a previously described method [15]. The CVA16-393-p3 was inoculated onto a fresh monolayer of MRC-5 cells in a 75-cm^2^ cell culture flask (Corning Costar, Bodenheim, Germany). After culturing for two passages, the master seed stock P5 was obtained. Two further passages in MRC-5 cells grown in a 225-cm^2^ cell culture flask yielded the working virus seed stock P7.

For subsequent passages of the P7 virus, MRC-5 cells at a 95% confluent monolayer were infected with P7 virus at a multiplicity of infection (MOI) of 0.5. The inoculated cells were cultured at 35 °C and monitored for CPEs daily. When 90-95% of the cells showed typical CPEs, the supernatants were harvested. The working virus seed P7 was consecutively passaged for 15 times in MRC-5 cells. For evaluation of the genetic stability, at the end of every five passages, namely, the 7th, 12^th^, 17th and 22^nd^ passages (CVA16-393-p7, CVA16-393-p12, CVA16-393-p17, CVA16-393-p22, respectively) were subjected to the nucleotide sequence analysis of the VP1 gene. The antigenic stability of the four different passages was evaluated by neutralization assays using anti-sera raised in rabbits. Briefly, rabbits (2-2.5 kg) were immunized with a CVA16 inactivated vaccine produced by the CVA16-393-p17 strain, and boosted one week later. The anti-sera were collected one week after the booster immunization.

### Growth kinetics assay

MRC-5 cells in a 95% confluent monolayer were infected with the P7 strain at an MOI of 0.1, 0.5 or 1.0. Each MOI was repeatedly inoculated five times. One hour post-adsorption at 37 °C, the viral inocula were removed. The cells were washed with phosphate-buffered saline (PBS), and incubated with 30 ml of virus growth medium (MEM containing 3% FBS) in an incubator at 35 °C. At each time point (24, 36, 48, 60, 72 and 84 h), flasks with the three different MOIs were harvested and stored at −80 °C. All the flasks containing the culture medium and cells were subjected to three freeze-thaw cycles. The supernatant was harvested by centrifugation at 2000 × g (Beckman, CA, USA) for 20 min and stored at −80 °C for further use.

### Virulence of CVA16-393 strains in gerbils

The virulence of the CVA16-393-p7 and CVA16-393-p22 strains was evaluated based on lethality in 21-day-old gerbils (*n* =  5–9 for each group, 17-19 g for each gerbil). The viruses were inoculated into gerbils intraperitoneally at a dose ranging from 10^1.0^ to 10^3.0^ TCID_50_ in a volume of 100 μl. The gerbils were monitored daily for clinical signs over a 20-day period. The grade of clinical disease was scored as follows: 0, healthy; 1, ruffled hair, hunchbacked or reduced mobility; 2, limb weakness; 3, paralysis in one limb; 4, paralysis in both limbs or deep lethargy; and 5, death. The mortality rate was calculated using Microsoft Excel 2007, and the 50% lethal dose (LD_50_) was calculated by the Reed and Muench method [13].

### Preparation of inactivated whole-virus CVA16 vaccine

The CVA16-393-p7 strain was inoculated onto a 95% confluent monolayer of MRC-5 cells, and incubated at 35 °C for 2–3 days. When more than 90% CPEs was observed, the infected cells and culture supernatant were harvested by three freeze–thaw cycles and centrifuged at 2000 × g for 20 min to remove the cellular debris. The supernatant, which was mixed with 37% formaldehyde solution (Sigma-Aldrich, MO, USA) at a volume ratio of 2500:1 and incubated at 37 °C for 5–6 days for virus inactivation, was subsequently filtered through a 0.22-μm syringe filter (Millipore, MA, USA). No live virus was detected during three successive blind passages on Vero cells. The inactivated supernatants were concentrated by ultrafiltration using a 100 kDa NMWC membrane (GE Healthcare, MA, USA), and purified by a Sepharose Fast Flow 6 gel column to prepare the inactivated CVA16 vaccine. The vaccines were stored at 4 °C for further use.

### Antigen assay for inactivated CVA16 vaccine

The antigen titre of the inactivated CVA16 vaccine was detected by an enzyme-linked immunosorbent assay (ELISA) as described below. Two-fold serial dilutions of the vaccine were transferred to the ELISA plates (Corning, NY, USA), and incubated for 2.5 h at 37 °C. After three washes, 100 μl of diluted rabbit anti-CVA16 sera (1:2000) was added and incubated at 37 °C for 60 min. Horseradish peroxidase-conjugated anti-rabbit antibody (1:5000, Sigma Aldrich, MO, USA) was added to each well sequentially. After incubation for 1 h at 37 °C, the optical density was measured at 450 nm. Values over 0.1 were regarded as positive, and the maximum dilution still showing a positive reaction was 1:400. In the inactivated vaccine, the concentration of the CVA16 antigen at this maximum dilution (1:400) was calculated as 4000 U/ml. Aluminum hydroxide (General Chemical LLC, NJ, USA) was used as the adjuvant at a final concentration of 0.5 mg/ml. PBS containing 0.5 mg/ml of aluminum hydroxide was used as the negative control.

### Immunization and neutralizing antibody assay

In this study, gerbils (aged 30 days) were randomly divided into three experimental groups that were immunized intraperitoneally with two doses of the formaldehyde-inactivated CVA16 vaccines at intervals of one, two, or four weeks. Each group of gerbils, which were randomly divided into four subgroups (5 gerbils per group), were immunized with a primary vaccine dose at 400, 100, 50 U, or a negative control. The gerbils were boosted with the same dose at each time interval. Blood samples were collected from the gerbils at 1, 3, 5 and 8 weeks after the second immunization, and the neutralizing antibody titre (NAT) was detected by a plaque reduction neutralization test (PRNT) [15]. Briefly, sera were heat-inactivated at 56 °C for 30 min, and two-fold serial dilutions of the serum samples in MEM were mixed with an equal volume of 200 × TCID_50_ of CVA16-194. After incubation for 2 h at 37 °C, the serum-virus mixture was added to Vero cells and incubated for 1 h at 37 °C. Then, the medium was removed, and the cells were overlaid with 0.4% nutrient agarose for 2 days. The second overlay containing neutral red (Sigma, Darmstadt, Germany) was added on day 3, and the plaques were counted on day 4. The NAT of the antiserum, which yielded a 50% reduction of the control’s plaque count (PRNT_50_), was calculated by the Reed-Muench method.

### Neutralizing antibody response to different CVA16 strains

Ten 30-day-old gerbils (female, 25-28 g) were immunized intraperitoneally with two doses of the CVA16 vaccines (100 U) at a one-week interval. PBS containing the aluminum hydroxide adjuvant (0.5 mg/ml) was used as the negative control. Blood samples were collected one week after the booster immunization. The NATs against five different CVA16 strains (11203SD, 10246, TJ-224, 40812TXT and CA16-194) were determined by the PRNT method.

### IFN-γ-specific enzyme-linked immunospot (ELISPOT) assay

Eight-week-old, female BALB/c mice were separated into the immunized and control groups (n =  5 for each group). The mice in the immunized group were vaccinated with 100 U of CVA16 vaccine and boosted on Day 7, and the mice in the control group were administered with PBS containing 0.5 mg/ml of aluminum hydroxide. Spleens were collected on 28 days post-immunization, and teased apart into single splenocyte suspensions. Splenocytes (5×10^5^ cells/well) were cultured in an ELISPOT plate for IFN-γ detection according to the manufacturer’s instructions (BD Biosciences, San Jose, NJ, USA). The CVA16 antigen (10 U/well) was used as the stimulant in the assay. Spot-forming cells (SFCs) were imaged with a ChemiDoc XRS+ imaging system (Bio-Rad, CA, USA), and analyzed using Quantity One software.

### Immunization and viral challenges

Gerbils (aged 7 days, weighting 6–7 g) were divided into five groups according to the five challenge strains. Each group contained five sub-groups (n = 5 for each sub-group). The gerbils in each sub-group were immunized intraperitoneally with 0.1 ml of the CVA16 vaccine at four-fold serial dilutions (100, 25, 6.25 or 1.56 U/gerbil). PBS containing the aluminum hydroxide adjuvant (0.5 mg/ml) was used as the negative control. All gerbils were boosted seven days later. One week after the booster immunization, both the immunized and control gerbils were challenged intraperitoneally with 100 × LD_50_ of the 11203SD, 10246, TJ-224, 40812TXT and CA16-194 strains. The gerbils were monitored daily for clinical signs for 20 days. The LD_50_ values of these strains in 21-day-old gerbils were determined to equal TCID_50_ values of 10^1.0^, 10^1.5^, 10^3.25^, 10^1.5^ and 10^1.5^.

### Histopathological analysis

Seven days after lethal challenge in the 1.56 U vaccine-immunized gerbils, the muscle and brainstem tissues of both healthy and diseased gerbils were collected immediately after anesthetization, fixed in 10% formalin for 4 days, dehydrated through ethanol gradients, and embedded in paraffin. The sections were sliced, mounted on poly-L-lysine-coated slides, and stained with hematoxylin and eosin or with Nissl. The score for the histological damage of muscle was assessed by measurements of the inflammatory cell infiltration and degeneration of the muscle fibres (0: normal; 1: mild; 2: moderate; and 3: severe) [16]. The histopathological score for the damage to brainstem was estimated based on measurements of the inflammatory cell reaction and lesions of the neurocytes (0: normal; 1: mild; 2: moderate; and 3: severe).

### Statistical analysis

All the data were analyzed using GraphPad Prism 8.0 or Microsoft Excel 2007 software. The Student’s t-test was used for comparisons between two groups, and *p *< 0.05 was considered as statistical significance.

## Results

### Phylogenetic analysis

**Figure 1.**
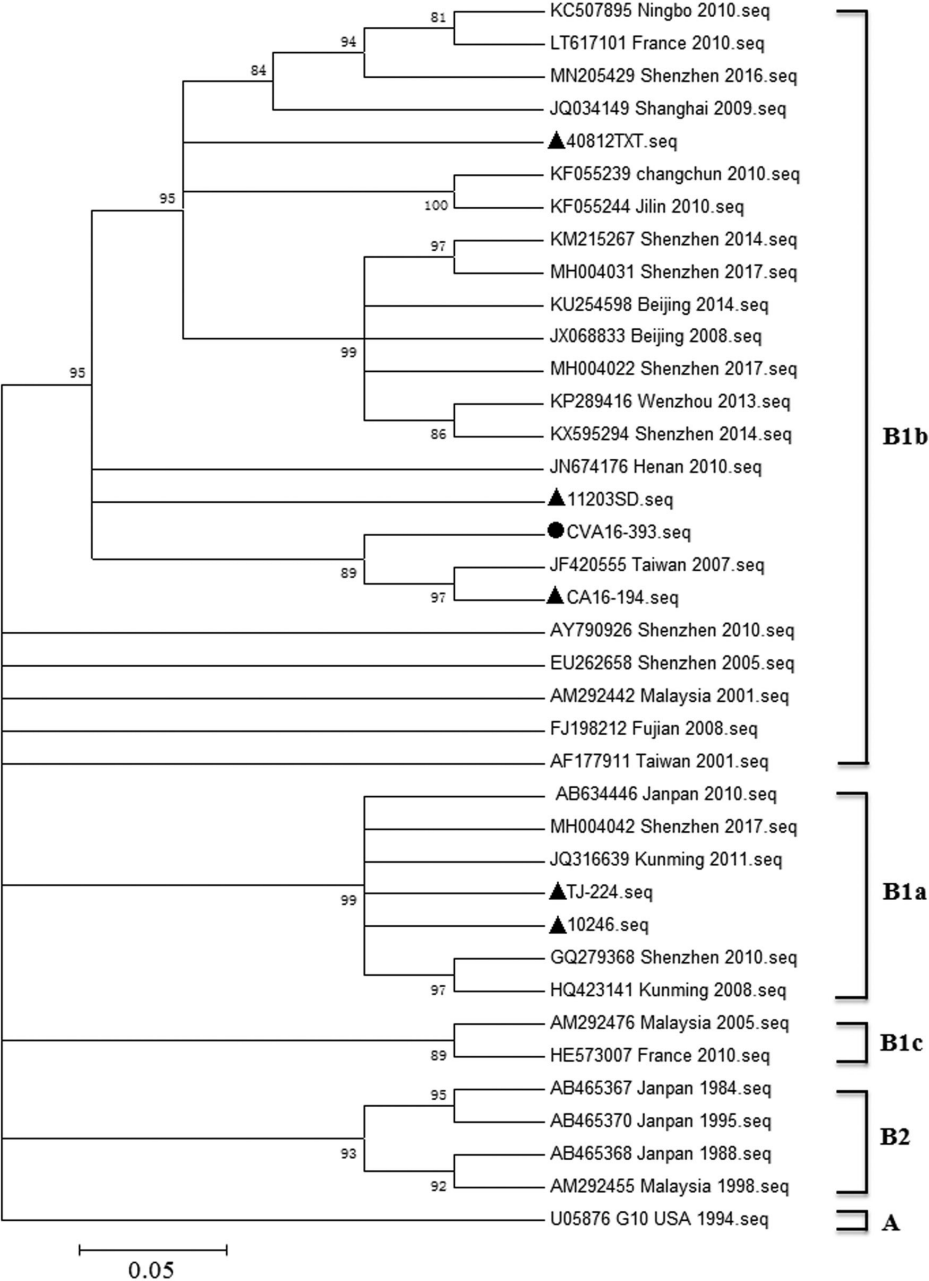
Phylogenetic tree based on the entire VP1 genome of 38 representative strains. The neighbor-joining method was used to construct the phylogenetic tree, and the bootstrap values (%) for 1000 replicates were calculated and shown at the nodes of major clades. The CVA16-393 sequence was marked with a circle (●), while the five sequences (11203SD, 10246, TJ-224, 40812TXT and CA16-194) used in this study were marked with a triangle (▴). The other 32 reference sequences were available in the GenBank database. MEGA 5.1 software was used in this study.

To determine the genotype of CVA16-393, a total of 38 sequences including CVA16-393 and the five strains (11203SD, 10246, TJ-224, 40812TXT and CA16-194) used in this study, were selected for comparison analysis (Figure 1). A phylogenetic tree was constructed based on the complete VP1 sequences (2448-3338 nucleotides) using the neighbor-joining method. The 32 reference strains from the Asia-Pacific region were classified into the genotypes or subgenotypes A, B1a, B1b, B1c and B2. The CVA16-393, as well as 40812TXT, 11203SD and CA16-194 strains, were grouped into the B1b subgenotype, whereas the 10246 and TJ-224 strains were grouped into the B1a subgenotype. CVA16-393 shared 92.7-97.0% nucleotide identity with other strains of the B1b subgenotype, and 89.1-91.8% nucleotide identity with the strains of the B1a subgenotype.

### Growth characteristics of CVA16-393 strains

**Figure 2.**
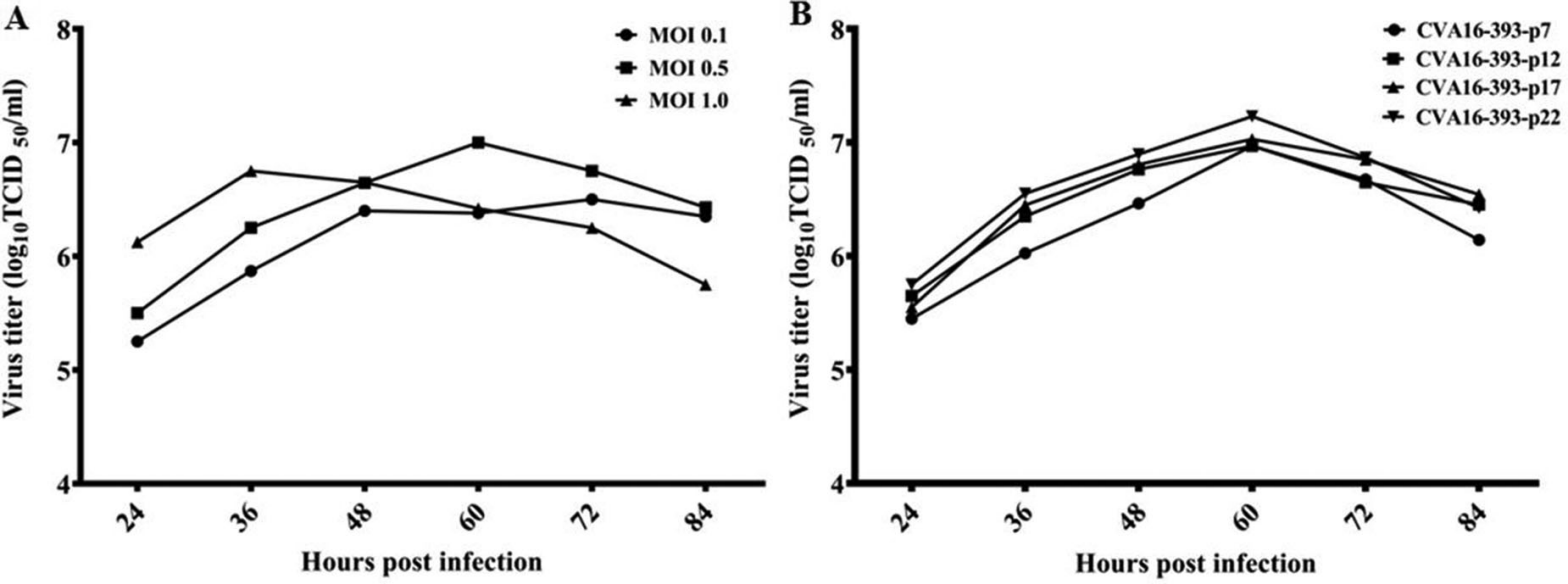
Replication kinetics of CVA16-393-p7 and growth curves of CVA16-393 at various passages in MRC-5 cells. (A) The viral titres of CVA16-393-p7 were determined by a TCID_50_ assay at 24, 36, 48, 60, 72 and 84 h post-infection at MOIs of 0.1, 0.5 and 1.0. (B) Cells were infected with 7th, 12^th^, 17th, and 22^nd^ passages (CVA16-393-p7, CVA16-393-p12, CVA16-393-p17, CVA16-393-p22) of CVA16-393 at an MOI of 0.5. The culture supernatants were collected at 24, 36, 48, 60, 72 and 84 h post-infection, and the virus titres were determined and shown as TCID_50_/ml.

To investigate the growth properties of the working virus seed stock CVA16-393-p7, MRC-5 cells were infected with various MOIs (0.1, 0.5 and 1.0). At a low MOI of 0.1, CVA16-393-p7 exhibited retarded growth kinetics and took approximately 72 h to reach the peak virus titre in the supernatant cultures (Figure 2A). However, at a high MOI infection (MOI = 1.0), the virus titres peaked at 36 h post-infection and then declined (Figure 2A), indicating of cell aging and rapid decay of viral viability. The time to reach the peak viral titre appeared to be inversely related to the MOI under the same culture conditions. At an MOI of 0.5, the peak virus titre was detected at 60 h post-infection and was approximately 10^7.0^ TCID_50_/ml_,_ which was significantly higher than the values obtained at other MOIs (0.1 and 1.0).

### Stability of CVA16-393

Viruses may change during prolonged culture in cells. As such, we determined the growth and antigenic stability of CVA16-393 after serial passages in MRC-5 cells. We selected the 7th, 12th, 17th, and 22nd passages of CVA16-393 (CVA16-393-p7, CVA16-393-p12, CVA16-393-p17 and CVA16-393-p22, respectively) to assess their growth stability in MRC-5 cells. The virus titres shown as TCID_50_/ml were measured at 24, 36, 48, 60, 72 and 84 h post-infection at an MOI of 0.5 (Figure 2B). Similar growth kinetics were obtained for all four passages. No significant difference in viral titres was detected among the four passages of CVA16-393 in MRC-5 cells. All the peaks of virus titre occurred at 60 h post-infection. The antigenic stability of the four different passages was evaluated by neutralization assays using anti-sera raised in rabbits, which were immunized with a CVA16 inactivated vaccine produced by the CVA16-393-p17 strain. The geometric mean titres (GMTs) for the CVA16-393-p7, CVA16-393-p12, CVA16-393-p17 and CVA16-393-p22 strains were 251.19, 354.81, 317.56 and 331.25, respectively (Table 1), indicating no significant changes in the antigenic properties.

**Table 1.** The cross-neutralization test for various CVA16-393 passages.

Virus passages	CVA16-393-p7	−p12	−p17	−p22
The geometric mean titre	251.19	354.81	317.56	331.25

**Antisera were raised in rabbits**

**Figure 3.**
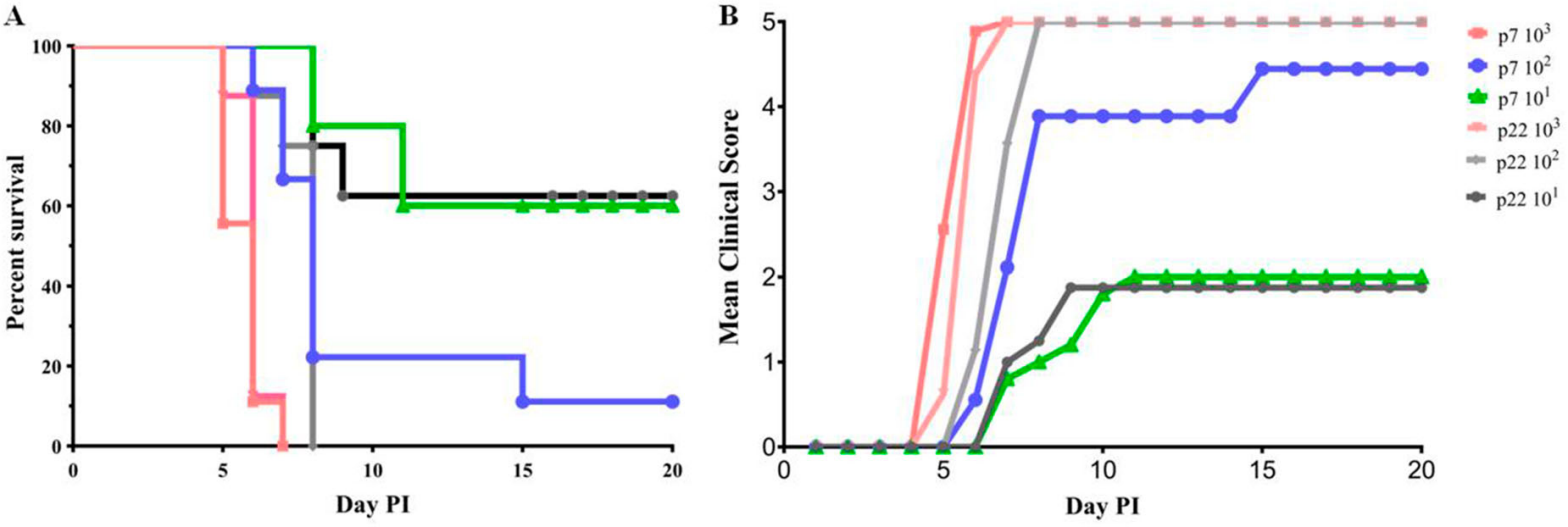
Survival rate and severity of disease in the 7th and 22^nd^ passages of CVA16-393-infected gerbils. (A) Survival curves for groups of 21-day-old gerbils (n = 5-9) when infected with the 7th and 22^nd^ passages (p7 and p22) of CVA16-393 at a TCID_50_ of 10^1^ to 10^3^. (B) Mean clinical scores for 21-day-old gerbils infected with the 7th and 22nd passages of CVA16-393 at a TCID_50_ of 10^1^ to 10^3^.

To assess the virulence of 7th and 22^nd^ passages of CVA16-393, 21-day-old gerbils were inoculated with a virus titre ranging from 10^1.0^ to 10^3.0^ TCID_50_. Intraperitoneal inoculation with 10^3.0^ TCID_50_ of the CVA16-393-p7 and CVA16-393-p22 strains in gerbils resulted in 100% mortality (Figure 3A). Clinical symptoms first appeared at 5 days post-infection, and all gerbils died within 7 days (Figure 3B). Gerbils inoculated with the CVA16-393-p7 and CVA16-393-p22 strains at a titre of 10^2.0^ TCID_50_ resulted in 88.89% (8/9) and 100% (9/9) mortality, respectively (Figure 3A). When the inoculation dose was reduced to 10^1.0^ TCID_50_, the survival rates of the infected gerbils in the two groups were 60.0% and 62.5%, respectively (Figure 3A). The LD_50_ of the CVA16-393-p7 strain was 1.60 × 10^1.0^ TCID_50_, while that of the CVA16-393-p22 strain was 1.59 × 10^1.0^ TCID_50_. These results indicated that there was no significant difference in virulence between the 7th and 22^nd^ passages of CVA16-393.

### Dynamic profile of the neutralizing antibody response in gerbils and the IFN-γ response in BALB/c mice

**Figure 4.**
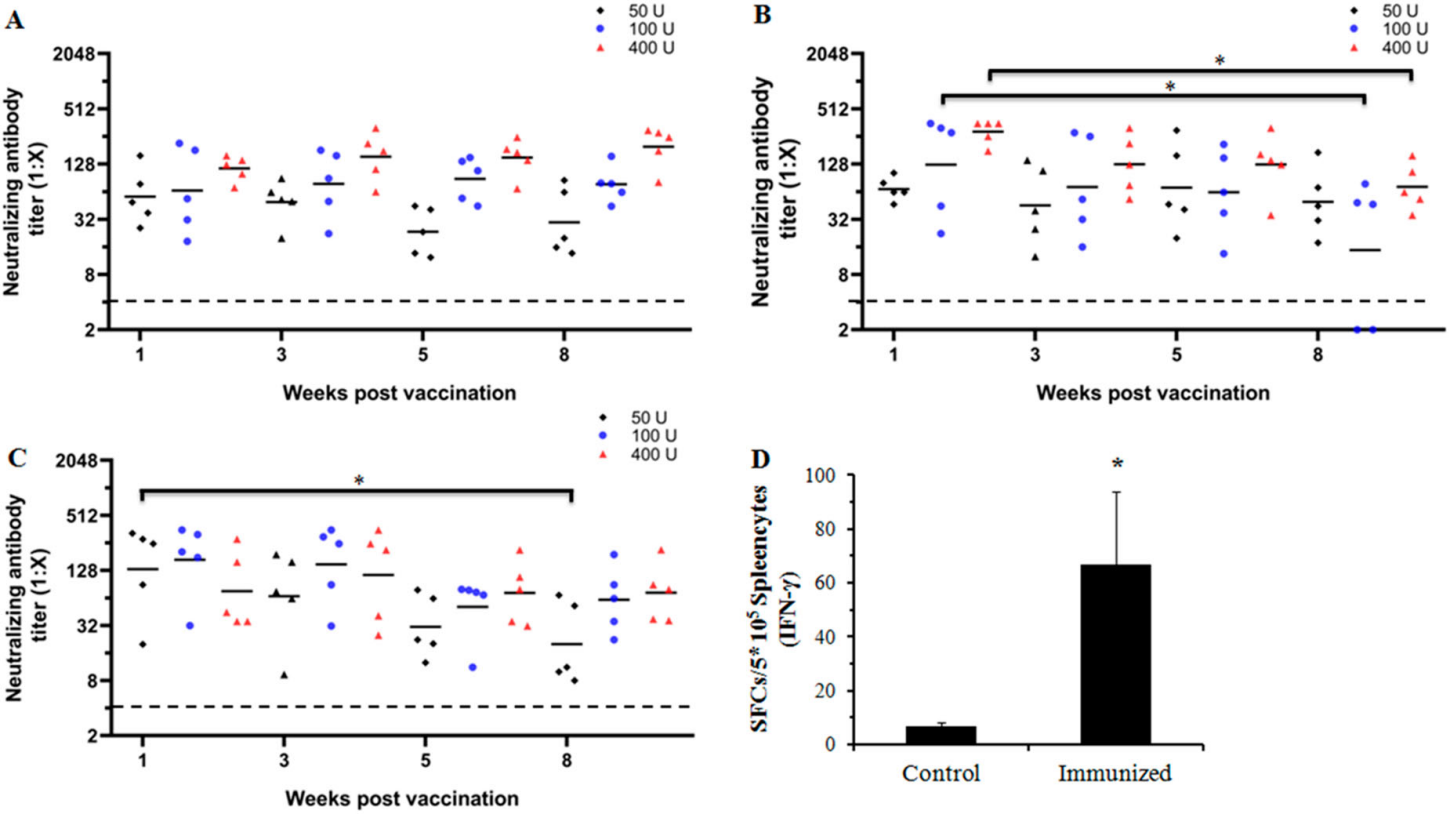
Dynamic profile of the neutralizing antibody response elicited by three CVA16 vaccine doses at different immunization intervals and the IFN-γ response in BALB/c mice. Gerbils were immunized with 50, 100 U/0.1 ml or 400 U/0.1 ml of the inactivated CVA16 vaccine, and received a booster vaccination at one- (A), two- (B), and four-week (C) intervals. Blood samples were collected at 1, 3, 5 and 8 weeks after the booster immunization. The dotted lines indicated the detection limit of this assay. (D) Eight-week-old BALB/c mice (n =  5 each group) were immunized with 100 U of CVA16 vaccine and boosted one week later. PBS containing 0.5 mg/ml of aluminum hydroxide was used as the control. Four weeks after immunization, the ELISPOT assay was performed to measure the IFN-γ response of the splenocytes. *Significant difference between the two groups (*p* < 0.05).

To investigate which immunization procedure could induce a high level and long-lasting neutralizing antibody response, 30-day-old gerbils were immunized with the CVA16 vaccine at doses ranging from 50 U to 400 U (n = 5 each group). A booster dose was administered after 1, 2, or 4 weeks, and the neutralizing antibody responses were subsequently determined at 1, 3, 5 and 8 weeks after the booster immunization. When the booster dose was administered at a one-week interval (Figure 4A), the GMTs of the neutralizing antibody were 56.6, 65.7 and 114.8 in the 50, 100 and 400 U groups one week after the second immunization. The NATs were induced in a dose-dependent manner, and could last for at least an eight-week monitoring period in different dose groups. In contrast to the two- or four-week-interval booster groups (Figure 4B and 4C), the NATs were peaked at the first week after booster vaccination, sustained for a short period, and waned subsequently. Eight weeks after immunization with the dose of 400 U, the GMTs of the neutralizing antibody were 72.3 and 73.1 in the two- and four-week-interval groups, much lower than 198.2 in the one-week-interval group. These results indicated that a booster dose after one week could induce an effective neutralizing antibody response in a dose-dependent manner.

To verify the cell mediated immunity induced by the vaccine, eight-week-old BALB/c mice (n =  5 each group) were immunized with 100 U of CVA16 vaccine and boosted one week later. An ELISPOT assay was performed to measure the IFN-γ response of the splenocytes. As shown in Figure 4D, the IFN-γ response in the immunized group was markedly higher than that in the control group, indicating a cellular immune response in the CVA16 vaccine-immunized mice.

### Antibodies induced by the CVA16 vaccine efficiently neutralize diverse CVA16 subtype strains

**Figure 5.**
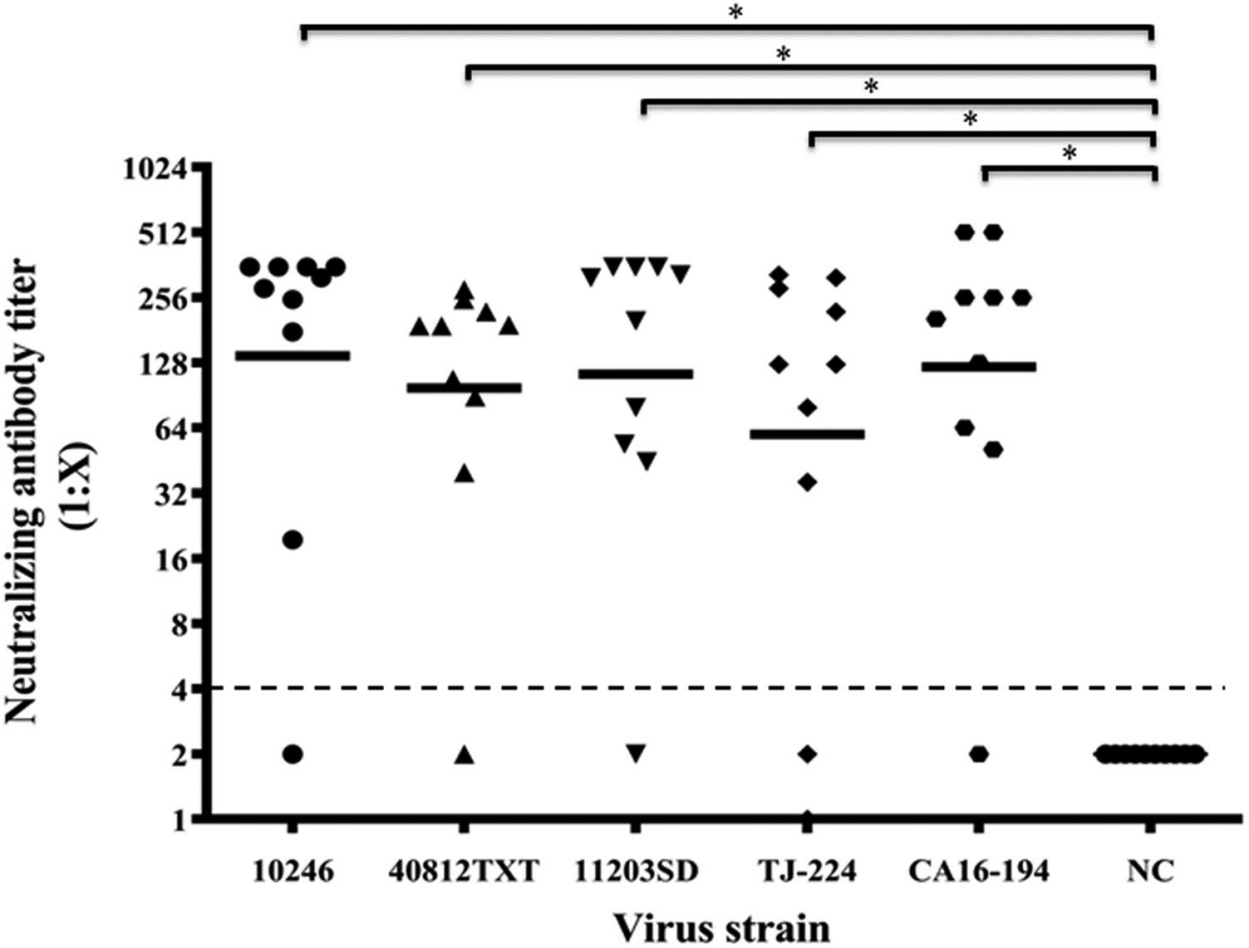
Neutralizing antibody titres against four CVA16 strains. The 30-day-old gerbils were immunized with 100 U/0.1 ml of the CVA16 vaccine and boosted one week later. Seven days after the second dose, the NATs of sera were determined by 100× TCID_50_ of five CVA16 strains (10246, 40812TXT, 11203SD, TJ-224 and CA16-194). Each group included 10 gerbils. PBS containing 0.5 mg/ml of aluminum hydroxide was used as the negative control. The dotted lines indicated the detection limit of this assay. *Significant difference between the two groups (*p* < 0.05).

To measure the efficacy of neutralizing antibodies elicited by the CVA16-393 immunogen, five strains (10246, 40812TXT, 11203SD, TJ-224 and CA16-194) representing two subtypes of CVA16 (B1a and B1b) were used. Gerbils were immunized with 100 U of the CVA16 vaccine, and the NATs of sera were measured against each strain. As shown in Figure 5, the GMTs of the neutralizing antibodies for the 10246, 40812TXT, 11203SD, TJ-224 and CA16-194 strains were 137.8, 97.8, 113.4, 64.1 and 122.3, respectively. The highest NAT of 512 was found in the CA16-194 strain group, and the neutralizing antibody seroconversion rate was over 80% in each group. These results indicated that antibodies induced by the CVA16 vaccine could neutralize both the B1a and B1b subtypes of the CVA16.

### Cross-protection against CVA16 in immunized gerbils

**Figure 6.**
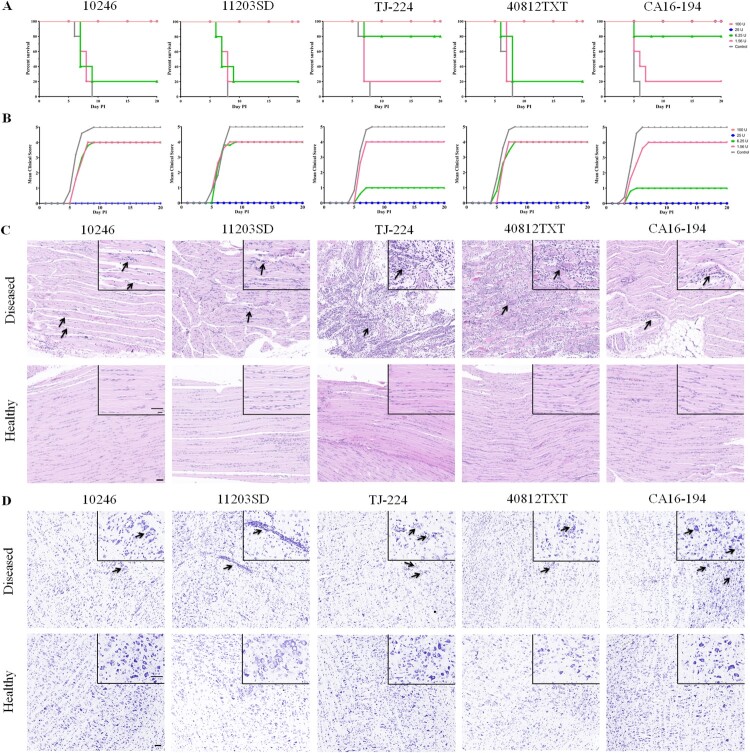
Evaluation of the cross-protection elicited by CVA16 vaccines in gerbils. Gerbils (aged 7 days) were immunized intraperitoneally with 0.1 ml of the CVA16 vaccine at four-fold serial dilutions (100, 25, 6.25, or 1.56 U/gerbil) and boosted one week later. One week after the booster immunization, gerbils were challenged intraperitoneally with 100 × LD_50_ of strains 11203SD, 10246, TJ-224, 40812TXT and CA16-194. (A) Survival curves for the challenged gerbils (*n* = 5). (B) Mean clinical scores of the challenged gerbils (*n* = 5). PBS containing the aluminum hydroxide adjuvant (0.5 mg/ml) was used as the negative control. The gerbils were monitored for clinical signs for 20 days. Seven days post-infection, muscle and brainstem tissues were collected from both the healthy and diseased gerbils belonging to the 1.56 U vaccine-immunized sub-groups. (C) Representative images of hematoxylin and eosin stained muscle tissues from both the healthy and diseased gerbils. The arrows indicated the inflammatory cell infiltration, and the degeneration of muscle fibres in the muscle tissue of the diseased gerbils. Bar: 50 μm. (D) Seven days post-infection, representative images of Nissl stained brainstem tissues were collected from healthy and diseased gerbils. The arrows indicated the neuronophagia, partial loss of Nissl’s body, perivascular infiltration by inflammatory cells, and swelling of neurocytes in the brainstem tissue of the diseased gerbils. Bar: 50 μm.

A gerbil model was used to evaluate the cross-protective effects elicited by the CVA16 vaccine. Gerbils (aged 7 days) were divided into five groups according to the challenge strains, and each group included five sub-groups (n = 5 for each sub-group). The gerbils were immunized with different CVA16 vaccine doses ranging from 1.56 to 100 U on Day 0 and 7. They were subsequently challenged with 100× the LD_50_ of 10246, 11203SD, TJ-224, 40812TXT and CA16-194 strains on Day 14. The vaccine provided protection against lethal challenges with all five CVA16 strains in a dose-dependent manner in gerbils (Figure 6A). When the immunization dose was higher than 25 U, all the gerbils could be protected. When the immunization doses were 6.25 or 1.56 U, the survival rates were reduced to 20-80% (Figure 6A). The diseased gerbils in 6.25 and 1.56 U sub-groups of each group all died within nine days after lethal challenge, whereas no clinical symptoms were observed in healthy gerbils of the corresponding sub-groups (Figure 6B).

We also analyzed the pathological changes in muscle and brainstem tissues of healthy and diseased gerbils of the 1.56 U sub-groups. As shown in Figure 6C, seven days after lethal challenge with the 10246, 11203SD, TJ-224, 40812TXT and CA16-194 strains, nearly no obvious lesion or inflammatory cell infiltration was found in the muscle tissue of the 1.56 U vaccine-immunized healthy gerbils. However, moderate or severe inflammatory cell infiltration and degeneration of muscle fibres were found in the 1.56 U vaccine-immunized diseased gerbils. The muscle tissues of the diseased gerbils were given histopathological scores of 2, 4, 6, 6 and 3 by measurements of the inflammatory cell infiltration and degeneration of muscle fibres (Table 2). The histopathological scores given to the muscle tissues of the diseased gerbils were significantly higher than those of the healthy gerbils in the same 1.56 U sub-groups. Neuronophagia, partial loss of Nissl’s bodies, perivascular infiltration by inflammatory cells, swelling and even necrosis of neurocytes were found in the brainstem of diseased gerbils (Figure 6D). The histopathological scores of the brainstem tissue of diseased gerbils were 4, 5, 2, 3 and 6, which were also significantly higher than those of the healthy gerbils in the same sub-groups (Table 2). If the 1.56 U vaccine-immunized gerbils survived after lethal challenge with the five CVA16 strains, no clinical symptoms and nearly no pathological lesions of muscle and brainstem tissues were found in these gerbils. All results showed that the CVA16 vaccine produced from the CVA16-393 strain was able to provide effective protection against challenge from both the B1a and B1b subtypes of the CVA16 in gerbils.

**Table 2.** Histopathological scores of 1.56 U vaccine-immunized gerbil tissues.

Challenge strains	Muscle								Brainstem							
	Inflammatory cell infiltration		Degeneration of the muscle fibres			Total score (Max score: 6)			Inflammatory cell infiltration			Lesions of the neurocytes			Total score (Max score: 6)	
	Diseased	Healthy	Diseased	Healthy		Diseased	Healthy		Diseased	Healthy		Diseased	Healthy		Diseased	Healthy
10246	1	0	1	0		2*	0		2	1		2	0		4*	1
11203SD	2	0	2	0		4*	0		3	0		2	1		5*	1
TJ-224	3	1	3	0		6*	1		1	0		1	0		2*	0
40812TXT	3	1	3	0		6*	1		2	0		1	1		3*	1
CA16-194	2	1	1	0		3*	1		3	1		3	0		6*	1

(0: normal; 1: mild; 2: moderate; and 3: severe). Diseased: Seven days post lethal challenge, the gerbils exhibited clinical symptoms in the 1.56 U vaccine-immunized groups. Healthy: Seven days post lethal challenge, the gerbils exhibited no clinical symptoms in the 1.56 U vaccine-immunized groups. *Significant difference compared with the healthy group (*p* < 0.05).

## Discussion

For the inactivated vaccine development, a suitable vaccine strain candidate should be selected based upon not only a molecular epidemiology study and the immunogenicity of the strain, but also the growth ability and genetic stability of the virus in host cells [17,18]. In this study, in an effort to develop a high-growth and high-immunogenicity strain for vaccine development, we selected the CVA16-393 strain based on its growth characteristics and antigenic stability after serial passages in MRC-5 cells. It grew well in host cells and reached a high titre of 10^7.0^ TCID_50_/ml. Through phylogenetic analysis, the CVA-393 strain was grouped into the B1b subgenotype, a predominant type of CVA16 in China [1,19]. The VP1 gene region was critical for the immunogenicity of the CVA16 vaccine, as major neutralization epitopes were located in this area [20]. A sequencing analysis of at least 20 serial passages of CVA16-393 in MRC-5 cells showed that the VP1 region was genetically stable (Supplementary Table S1 and Figure S1). The CVA16 vaccine produced from the CVA16-393 strain could induce an effective neutralizing antibody response in gerbils. Furthermore, the CVA16 vaccine was also able to provide a cross-protection against lethal challenge from both B1a and B1b subtype strains from different regions of China in a gerbil model. Through an ELISPOT assay, a significantly higher IFN-γ response was found in the immunized BALB/c mice than the control. Therefore, the CVA16-393 strain, which exhibits potential advantages for vaccine development in MRC-5 cells, might be a suitable vaccine candidate strain.

Several CVA16 candidate vaccines, such as the virus-like particle (VLP) vaccine and adenoviral vector vaccine, were developed, and their validity was evaluated in animals [21,22]. These vaccines showed good immune responses in mice, and the maternal antibodies induced by these vaccines were able to protect neonatal mice from CVA16 challenge. However, as compared with the virus-like particle vaccine and the adenoviral vector vaccine, the inactivated vaccine has several advantages, such as their well-established regulatory standards and simple streamlining of research. As a result, inactivated vaccines can be developed more rapidly and licensed to meet the immediate demands for disease control and prevention [23]. Currently, mRNA vaccines have been widely used in humans since the outbreak of the COVID-19 pandemic [24]. Compared with the inactivated vaccines, mRNA vaccine could be developed more quickly because they do not require pathogen purification and cell culture [25]. However, as a new type of nucleic acid vaccine, although no risk of genetic mutagenesis and carcinogenesis theoretically exists, the long-term potential adverse effects of mRNA vaccines in a large human population should be considered.

A suitable cell culture system is important for the inactivated vaccine development. The human diploid cell culture system is a safe platform that is widely used in biopharmaceutical research, vaccine production, and some licensed protein therapeutics [26]. Most of the reported inactivated CVA16 vaccines, which induce effective immunity, were produced in Vero cells [27], but a few of them were obtained from the human diploid cell line KMB17 [28]. However, no CVA16 vaccine has yet been produced with MRC-5 cells. As a human diploid cell line, MRC-5 cells have been used in the production of several vaccines including rabies, MMR, varicella (chickenpox), hepatitis A, shingles, and polio vaccines [29]. MRC-5 cells grow slowly, and some viruses cannot propagate in this cell line. In our study, a clinical swab sample was inoculated on MRC-5 cells for virus isolation, and the isolated CVA16 strain was easily propagated in MRC-5 cells. During the first passage, CPEs were observed at an average of approximately 7 days post-infection (dpi). When the harvested culture supernatant was passaged continuously 4–5 times, the CPEs were observed at 2 to 3 dpi (data not shown). After serial passages, the clinically isolated strain CVA16-393 reached a peak titre of 10^7.0^ to 10^7.5^ TCID_50_/ml. Thus, it appeared that MRC-5 cells well supported the growth of CVA16 and might be an ideal platform for the CVA16 vaccine production.

A highly virulent virus strain is important for the development of an inactivated vaccine. Serial passaging in cell lines can lead to a decrease in virulence or even avirulent virus strains [30,31]. In our study, no significant decrease in virulence during serial passages was found through detection of the LD_50_ values of the 7th and 22nd passages of CVA16-393 in gerbils. The GMTs of the CVA16-393-p7, CVA16-393-p12, CVA16-393-p17 and CVA16-393-p22 strains were also not significantly changed by the use of anti-sera from rabbits. Combined with the similar growth rate of CVA16-393-p7, CVA16-393-p12, CVA16-393-p17 and CVA16-393-p22, we might conclude that the antigen of the CVA16-393 strain was stable and could be used for vaccine production. The growth kinetics and inoculum dose of the virus are important for vaccine production. When the MOI was 1.0, the virus titre peaked early and declined quickly. When the MOI was 0.1, the virus grew slowly and the corresponding virus titre at each time point was lower than that in other groups. When the MOI was 0.5, the virus titre reached the highest value of three groups and declined slowly. Therefore, an MOI of 0.5 might be the proper inoculum dose.

Although inactivated vaccines are considered more stable and safer than live vaccines, some inactivated vaccines are generally less immunogenic than their live counterparts. To measure the immunogenicity of the vaccine produced by the CVA16-393 strain, a cross-neutralization test was carried out using five strains representing the B1a and B1b subtypes of CVA16 isolated from four different regions in China. The results showed that sera from CVA16 vaccine-immunized gerbils could neutralize all five strains. Furthermore, in the cross-protection experiment, an immunization dose higher than 25 U could provide 100% protection against lethal challenge from five strains in gerbils. When the immunization dose reduced to 1.56 U, the gerbils that survived from lethal challenge were healthy and had no clinical symptoms, while the diseased gerbils belonging to the same 1.56 U sub-groups exhibited obvious clinical symptoms and all died finally. The severe HFMD patients infected by CVA16 usually exhibit nervous system lesions or neurological complications, indicating a neurological tropism for CVA16 [12]. We also found neuronophagia, swelling and even necrosis of neurocytes in the brainstem of the diseased gerbils in the 1.56 U sub-groups. However, nearly no lesions were found in the brainstem of the healthy gerbils in the same sub-groups, and the score for histopathological damage in the healthy gerbils was significantly lower than that in the diseased gerbils. No degeneration of muscle fibres and nearly no inflammatory cell infiltration were found in the muscle tissue of the healthy gerbils in the 1.56 U sub-groups. In the low dose vaccine-immunized groups, a certain proportion of gerbils (20-80%) could still be protected from lethal challenge, with nearly no pathological lesion in the muscle and brainstem tissues. The CVA16 vaccine produced by the CVA16-393 strain might be an effective vaccine candidate.

To date, several animal models have been developed to study the pathogenesis of the CVA16 virus and assess the CVA16 vaccines. The neonatal mouse model is a suitable animal model, however, the sensitive period for CVA16 is only one week [32]. With such a short sensitive period, active immunization assays for vaccine development could not be conducted because the entire immunization process usually requires more than one week [12]. Rhesus macaques can partially mimic the pathological process of CVA16 infection, but no effective immune response induced by the CVA16 infection is detected. A neutralizing antibody and the IFN-γ-specific ELISPOT response could be induced by an inactivated CVA16 vaccine in rhesus macaques. However, the immune response induced by the CVA16 vaccine or the CVA16 infection could not provide clinical protection against further infections [33]. It appeared that the failure to induce a protective immune response in rhesus macaques might be due to host factors. Compared with the mouse model, rhesus macaques are more expensive, and associated with increased ethical concerns and housing risks. Our facility limitations also restricted the use of rhesus macaques. Although rhesus macaques are closer to humans than other animal models, they might not be an appropriate CVA16 animal model for wide use. Gerbils are similar to mice and rats, and belong to the family *Muridae*. Compared with other rodents, gerbils are better suited for a variety of disease models, such as human ocular toxocariasis, and leishmaniasis [34,35]. Our previous study showed that gerbils, which could be easily infected with the CVA16 virus, could develop central nervous system and muscle damage, eventually resulting in death [12,13]. Twenty-one-day-old gerbils remained sensitive to CVA16, and the CVA16 vaccine triggered an effective immune response in this model. Although no commercial reagent is available for the IFN-γ-specific response detection in gerbils, they might still be appropriate for evaluating a CVA16 vaccine.

Vaccination strategies should consider both the high-level and long-lasting neutralizing antibodies. In this study, gerbils were immunized with the CVA16 vaccine twice in a one-, two-, or four-week interval at a dose ranging from 50 U to 400 U, and neutralizing antibody responses were subsequently detected after 1, 3, 5 and 8 weeks. In the two- and four-week booster groups, the NATs were peaked within the first week after the booster, and waned subsequently. In contrast to the one-week booster group, the NATs were induced in a dose-dependent manner and were maintained at a relatively high level for at least 8 weeks. Our results demonstrated that a one-week interval vaccination strategy might be a suitable choice for CVA16 immunization in this gerbil model.

Overall, we demonstrated that the CVA16-393 strain not only grew well and induced a high viral titre in MRC-5 cells, but also retained genetic stability and virulence characteristics for at least 20 passages. Additionally, the CVA16 vaccine produced by the CVA16-393 strain provided effective cross-protection in gerbils, and the CVA16-393 strain might be a suitable strain candidate for VA16 vaccine development.

## Supplementary Material

Supplemental Material
