# Supplementary material for: Development and evaluation of an inactivated coxsackievirus A16 vaccine in gerbils
Source: Emerg Microbes Infect. 2022 Aug 11;11(1):1994–2006. doi: 10.1080/22221751.2022.2093132 (PMC9377242; doi:10.1080/22221751.2022.2093132)

**Development and evaluation of an inactivated Coxsackievirus A16 vaccine in gerbils**

Yi-Sheng Sun^a*^, Yong Xia^a*^, Fang Xu^a^, Hang-Jing Lu^a^, Zi-An Mao^b^, Meng Gao^b^, Tian-Yuan Pan^c^, Ping-Ping Yao^a^, Zhen Wang^a^, Han-Ping Zhu^a^

^a^ Key Lab of Vaccine, Prevention and Control of Infectious Disease of Zhejiang Province, Zhejiang Provincial Center for Disease Control and Prevention, Hangzhou, China

^b^Zhejiang Pukang Biotechnology Co., LTD., China

^c^Department of General Medicine, The First Affiliated Hospital, Zhejiang University, Hangzhou, China

* These authors contributed equally to this work

Correspondence: Ping-Ping Yao, Email: [ppyao@cdc.zj.cn](mailto:ppyao@cdc.zj.cn); Zhen Wang, Email: wangzhen@cdc.zj.cn; Han-Ping Zhu, Email: hpzhu@cdc.zj.cn

| **Supplementary Table S1. The VP1 nucleotide sequences of CVA16-393** | |
| --- | --- |
| Position | The nucleotide sequences |
| 1-60 | GGGGATCCTA TTGCAGATAT GATTGACCAG ACTGTGAACA ATCAAGTGAA TCGCTCCTTG |
| 61-120 | ACTGCGTTGC AAGTACTACC TACAGCTGCC AACACTGAAG CAAGTAGTCA CAGATTGGGT |
| 121-180 | ACTGGTATTG TACCAGCACT ACAAGCCGCG GAGACAGGGG CGTCGTCTAA TGCTAGTGAC |
| 181-240 | AAGAACCTCA TTGAGACTAG ATGTGTGTTG AACCATCACT CCACACAGGA GACAGCCATT |
| 241-300 | GGGAATTTCT TTAGCCGTGC CGGTCTTGTC AGTATTATTA CAATGCCCAC CACAGGTACA |
| 301-360 | CAGAACACAG ATGGTTATGT AAATTGGGAC ATCGACCTGA TGGGATATGC TCAGCTGCGG |
| 361-420 | CGGAAATGCG AATTGTTTAC CTACATGCGT TTTGACGCTG AATTCACATT TGTCGTAGCC |
| 421-480 | AAACCCAATG GTGAGCTAGT CCCCCAATTA CTGCAGTACA TGTATGTCCC ACCAGGGGCT |
| 481-540 | CCGAAACCTA CCTCCAGAGA CTCGTTTGCC TGGCAGACTG CTACCAACCC ATCTGTGTTT |
| 541-600 | GTGAAAATGA CGGACCCACC AGCTCAAGTG TCAGTCCCCT TCATGTCACC AGCTAGTGCA |
| 601-660 | TACCAATGGT TTTATGATGG TTATCCCACC TTCGGAGAGC ACCTCCAAGC AAATGACTTG |
| 661-720 | GATTATGGCC AATGCCCGAA TAATATGATG GGCACTTTTA GCATTAGGAC AGTAGGGACT |
| 721-780 | GAGAAGTCAC CACACTCCAT TACCCTGAGG GTGTACATGA GAATCAAACA CGTTAGGGCA |
| 781-840 | TGGATCCCAA GACCTCTGAG AAATCAACCC TATTTGTTTA AGACCAACCC AAATTATAAA |
| 841-891 | GGAAATGATA TTAAGTGCAC CAGCACCAGT AGAGACAAGA TAACAACGTT G |

**Supplementary Figure S1.** The comparison of VP1 nucleotide sequences of CVA16-393-p3 to CVA16-393-p22. The software DNAMAN 8.0 was used to do the multiple sequence alignment.


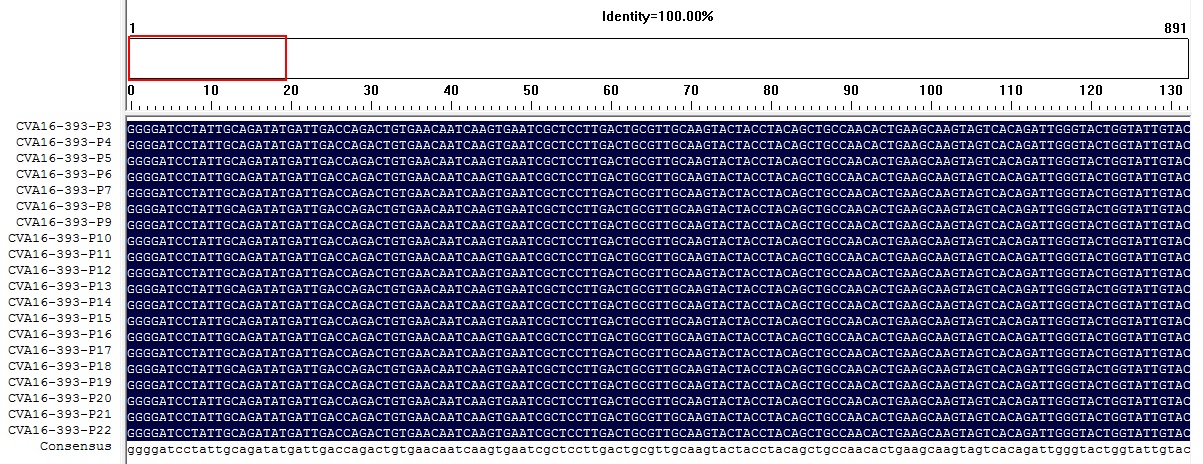

Supplement: Supplemental Material [file TEMI_A_2093132_SM6090.docx]
